# Basal hypersecretion of glucagon and insulin from palmitate-exposed human islets depends on FFAR1 but not decreased somatostatin secretion

**DOI:** 10.1038/s41598-017-04730-5

**Published:** 2017-07-05

**Authors:** H. Kristinsson, E. Sargsyan, H. Manell, D. M. Smith, S. O. Göpel, P. Bergsten

**Affiliations:** 1 Department of Medical Cell Biology, Uppsala University, BMC, Husargatan 3, Uppsala, Sweden; 2 Discovery Sciences, Innovative Medicines and Early Development Biotech Unit, AstraZeneca, Cambridge, UK; 3 AstraZeneca R&D Gothenburg, CVMD Bioscience, Gothenburg, Sweden

## Abstract

In obesity fasting levels of both glucagon and insulin are elevated. In these subjects fasting levels of the free fatty acid palmitate are raised. We have demonstrated that palmitate enhances glucose-stimulated insulin secretion from isolated human islets via free fatty acid receptor 1 (FFAR1/GPR40). Since FFAR1 is also present on glucagon-secreting alpha-cells, we hypothesized that palmitate simultaneously stimulates secretion of glucagon and insulin at fasting glucose concentrations. In addition, we hypothesized that concomitant hypersecretion of glucagon and insulin was also contributed by reduced somatostatin secretion. We found basal glucagon, insulin and somatostatin secretion and respiration from human islets, to be enhanced during palmitate treatment at normoglycemia. Secretion of all hormones and mitochondrial respiration were lowered when FFAR1 or fatty acid β-oxidation was inhibited. The findings were confirmed in the human beta-cell line EndoC-βH1. We conclude that fatty acids enhance both glucagon and insulin secretion at fasting glucose concentrations and that FFAR1 and enhanced mitochondrial metabolism but not lowered somatostatin secretion are crucial in this effect. The ability of chronically elevated palmitate levels to simultaneously increase basal secretion of glucagon and insulin positions elevated levels of fatty acids as potential triggering factors for the development of obesity and impaired glucose control.

## Introduction

Subjects with obesity have, in addition to elevated circulating insulin concentrations^1^, high glucagon levels at fasting^1, 2^. Although much attention has been on the raised insulin levels precipitating development of type 2 diabetes mellitus (T2DM)^3^, the high fasting glucagon levels have gained more and more attention as equally important for disease progression towards T2DM^4^. Under normal circumstances elevation of glucose has opposite effects on the secretion of insulin and glucagon from beta- and alpha-cells, this is paradoxical in T2DM patient were elevation of glucose stimulates both hormones^5^. Elevated glucagon levels trigger hepatic glucose output leading to higher insulin demand, which could thereby accelerate beta-cell stress and thus contribute to development of the disease^6^.

When identifying causes of the high fasting glucagon and insulin levels, circulating free fatty acids (FFAs) have been considered since they are elevated in obesity and such elevation is associated with an increased risk of developing T2DM^7^. Whereas independent relation between fasting FFAs and insulin levels has been confirmed *in vivo* ^8^, such relation has not been observed between fasting FFAs and glucagon. Causality between increased FFAs and rise in basal hormonal secretion has been addressed for insulin^9, 10^. With regard to effects of FFAs on glucagon secretion, there are reports of a stimulatory effect during acute exposure followed by a decline after long-term exposure^11–14^. How FFAs affect basal glucagon secretion from the human islet has not been defined, however. Long-chain FFAs including palmitate bind to free fatty acid receptor 1 (FFAR1/GPR40), which is highly expressed in the islets of Langerhans^15^. The receptor is expressed on beta-cells, where FFAR1 signaling is tightly coupled to mitochondrial respiration during palmitate exposure at high glucose concentrations^16^. Also, there is evidence that the receptor is expressed on alpha- and delta-cells and can affect glucagon secretion from rodent cells^17–19^. To what extent FFAR1 is involved in enhanced secretion of glucagon and insulin at fasting glucose concentrations in human islets has not been defined, however.

Secretion of glucagon and insulin at fasting could be further regulated by the paracrine effects e.g. the inhibitory effects of insulin on alpha-cells^20–22^ and somatostatin on both alpha- and beta-cells^23, 24^. Based on these observations a decrease in somatostatin secretion could contribute to enhanced secretion of both glucagon and insulin. To what extent such reduced somatostatin release is observed at fasting conditions, when glucagon and insulin secretion is increased, is not known, however.

Given the situation in obese children with concomitant elevation of both insulin and glucagon^1^ and elevated FFA levels at normal fasting glucose concentrations^25^, our aim was to examine how chronically elevated FFA levels affected glucagon, insulin and also somatostatin secretion from isolated human islets at fasting glucose concentrations with special focus on the role of FFAR1.

## Results

### Palmitate elevates basal islet glucagon and insulin secretion via FFAR1

**Figure 1:**
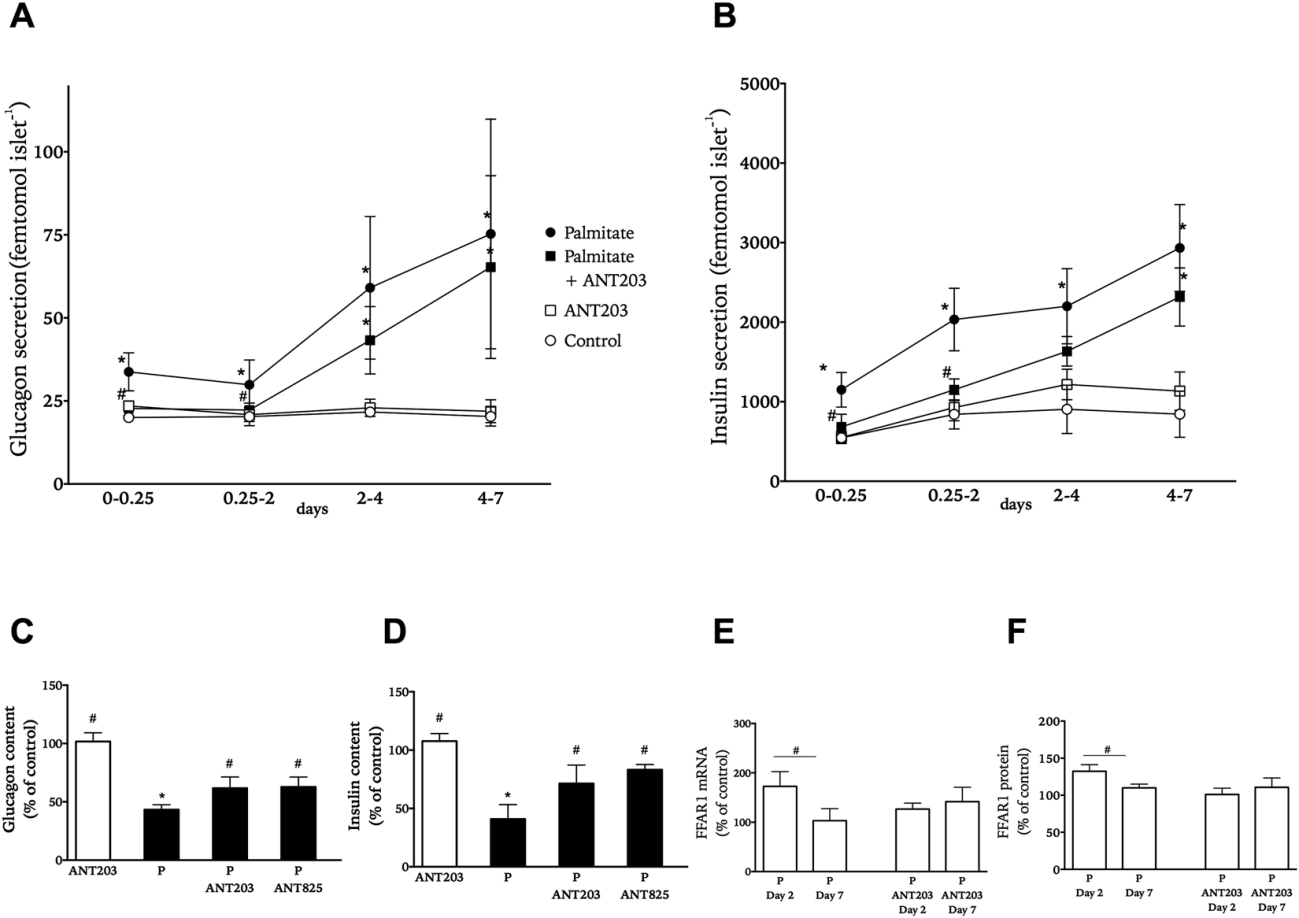
Effect of palmitate on accumulated glucagon (panel A) and insulin secretion (panel B) from isolated human islets cultured for 0.25 to 7 days at 5.6 mM glucose in the absence (white symbols) or presence (black symbols) of palmitate with and without FFAR1 antagonist ANT203. Cellular content of glucagon (panel C) and insulin (panel D) at the end of culture in palmitate-treated islets with and without FFAR1 antagonists ANT203 and ANT825 presented as % of the hormone content in islets receiving control treatment. Levels of FFAR1 mRNA (panel E) and protein (panel F) in palmitate-treated islets with and without FFAR1 antagonist ANT203. Transcript and protein levels presented as % of levels in islets receiving control treatment. Results show mean ± SEM from n = 3–5 experiments donor experiments. *P < 0.05 vs. control and ^#^p < 0.05 vs. palmitate.

To determine the effect of long-chain fatty acid palmitate on glucagon and insulin secretion at fasting glucose concentrations isolated human islets were cultured at 0.5 mM palmitate and 5.6 mM glucose. Both glucagon and insulin accumulation was elevated after 6 hours of culture as compared to control treatment and continuously increased during the culture period (Fig. 1A,B). Palmitate-treated islets had about 40–50% cellular glucagon and insulin content of that in control islets after 7 days of culture (Fig. 1C,D). To investigate if amplification of hormone secretion by palmitate involved FFAR1 activation we co-cultured islets exposed to palmitate with FFAR1 antagonist ANT203. In the presence of ANT203 glucagon and insulin release from palmitate-treated islets was reduced to levels close to those secreted from control islets during the first 2 days (Fig. 1A,B). For the remaining culture time, co-culture with ANT203 no longer statistically significantly lowered glucagon or insulin accumulation from islets. At the end of culture there was substantially higher hormone content in the palmitate plus ANT203 group compared with palmitate alone (Fig. 1A,D). Palmitate culture caused an initial increase in FFAR1 protein levels with subsequent relative decline in levels at the end of culture (Fig. 1E), whereas co-culture with ANT203 did not statistically significantly alter FFAR1 levels during culture (Fig. 1F).

### FFAR1-reduction counteracts palmitate-induced increase in basal islet hormone secretion

**Figure 2:**
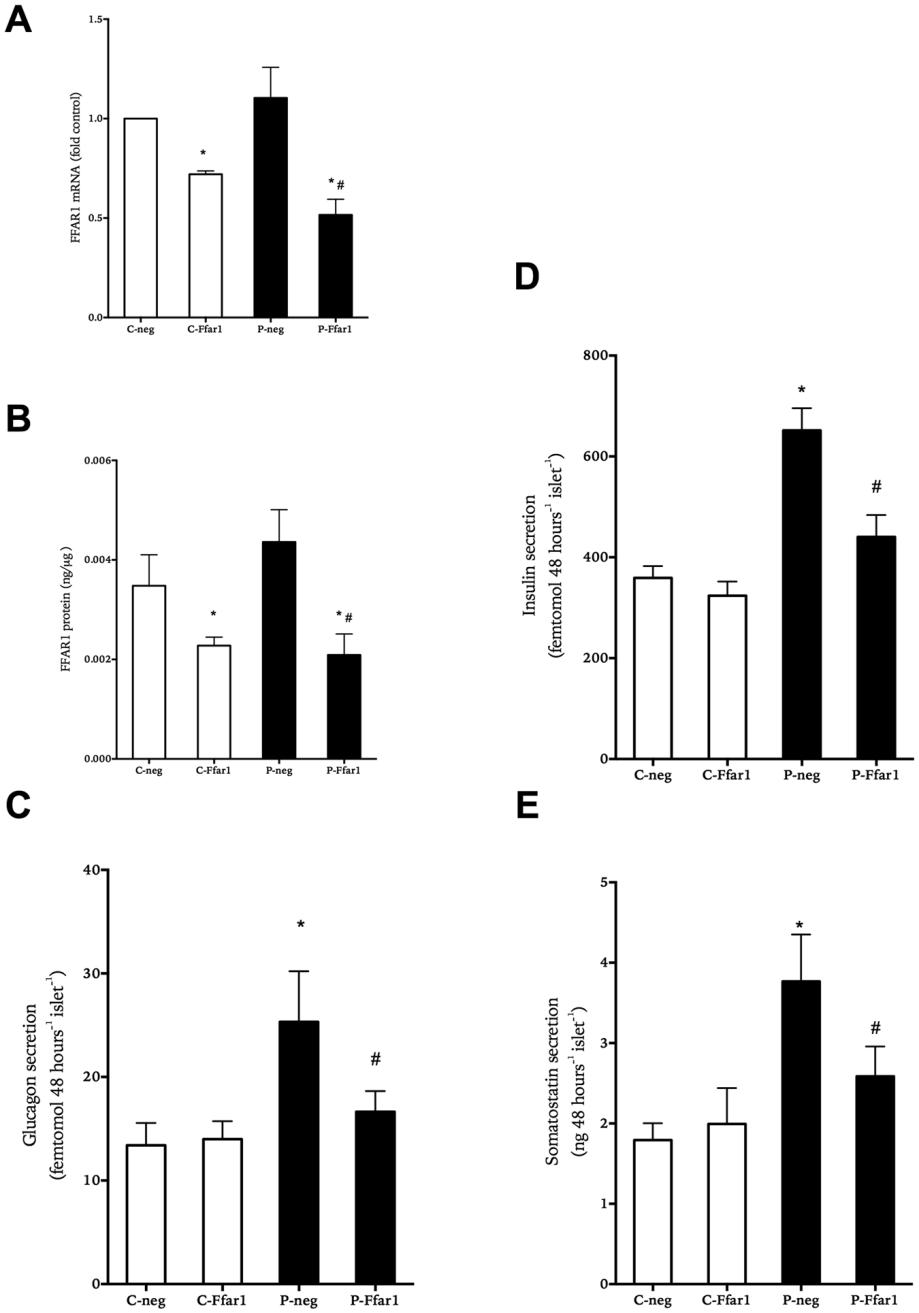
Effect of decreasing FFAR1 expression on accumulated hormone secretion from human islets cultured at 5.6 mM glucose in the absence (white bars) or presence of 0.5 mM palmitate (black bars) for 1 day. Relative FFAR1 mRNA levels (panel A) and total FFAR1 protein (panel B) at the end of culture as compared to non-mammalian target shRNA (neg) or shRNA targeting Ffar1 (Ffar1) treatment. Accumulated secretion of glucagon (panel C), insulin (panel D) and somatostatin (panel E) is shown. Results show mean ± SEM and are from n = 3–5 donor experiments. *P < 0.05 vs. control-neg, ^#^p < 0.05 vs. palmitate-neg.

In order to confirm the role of FFAR1 in modulating the effects of palmitate on basal islet hormone secretion, we reduced the expression of FFAR1 in isolated human islets. Expression of islet FFAR1 was significantly reduced by transfection with the shRNA for FFAR1 (Fig. 2A and B). In control islets exposed to palmitate for 2 days glucagon and insulin accumulation increased by around 50% and 2-fold, respectively, and somatostatin by 2-fold (Fig. 2B–D). In islets with reduced FFAR1 expression palmitate induced less accumulation of glucagon, insulin and somatostatin compared to islets with normal FFAR1 expression (Fig. 2C–E).

### Palmitate amplifies basal islet hormone secretion via FFAR1 and fatty acid metabolism

**Figure 3:**
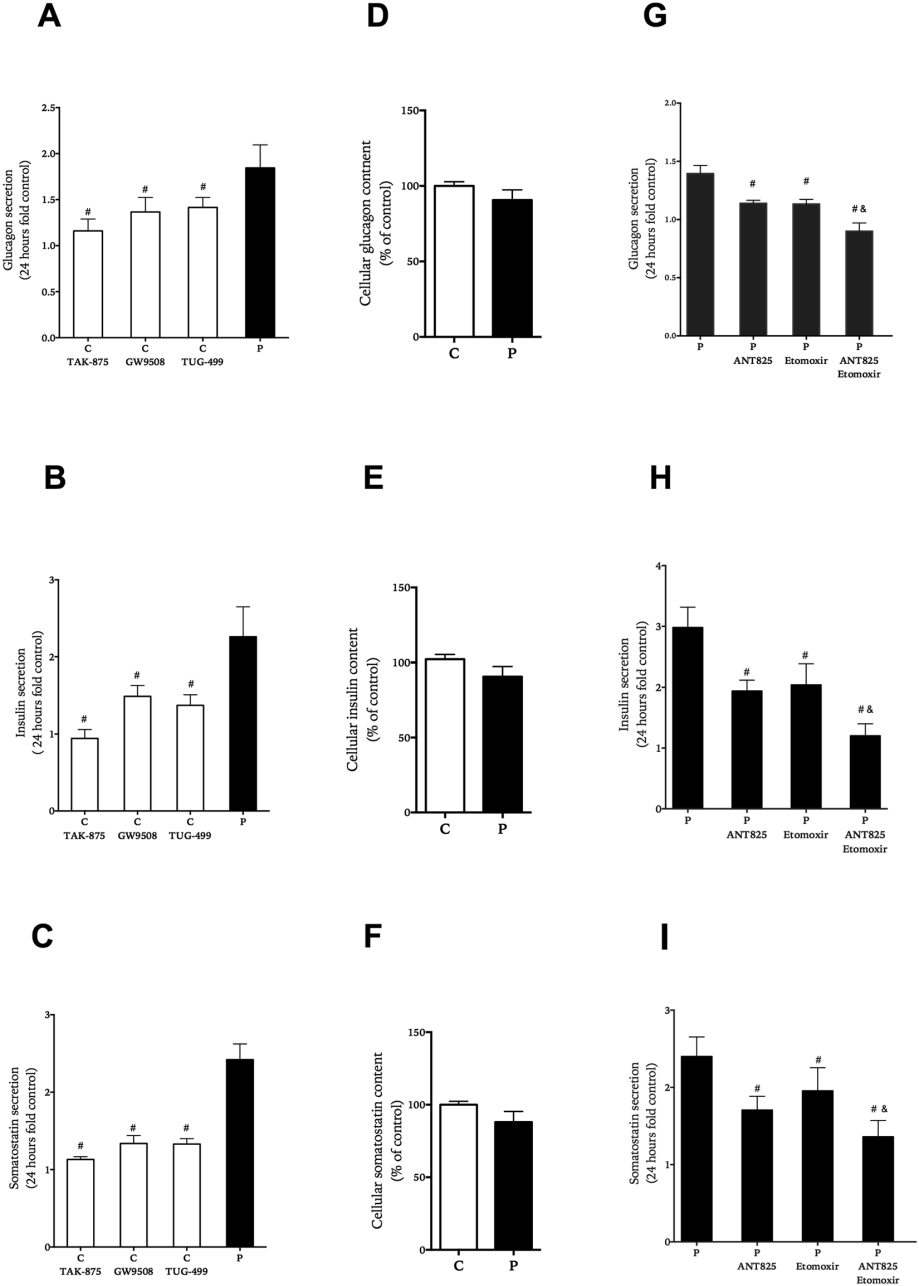
Effect of FFAR1 and fatty acid β-oxidation on basal glucagon (panels A,G), insulin (panels B,H) and somatostatin (panels C,I) secretion and content (panels D-F,F). Human islets were cultured for 1 day with or with out FFAR1 agonists; TAK-875, GW9508, TUG-499 in comparison to palmitate (black bars) (panel A–C), and with palmitate with and without ANT825 and/or etomoxir (panel G–I). Results show mean ± SEM and are from n = 5 donor experiments. ^#^P < 0.05 vs. palmitate, and & vs. palmitate + etomoxir.

Palmitate increased accumulation of glucagon by about 50% and insulin and somatostatin 2–3-fold from islets exposed to palmitate for 24 hours (Fig. 3A–C,G–I). After this culture period hormonal content was not statistically significantly changed between control and palmitate conditions (Fig. 3D–F) in contrast to what was observed for extended culture period (Fig. 1C,D). When synthetic FFAR1 agonists were included during culture of islets not exposed to palmitate the increase was moderate compared to the increase induced by palmitate, however. The finding that synthetic non-fuel agonists to FFAR1 had milder effects on insulin, glucagon and somatostatin release than palmitate (Fig. 3A–C) made us investigate the role of metabolism of the fatty acid. When islets were cultured with palmitate and FFAR1-antagonist ANT825 and/or inhibitor of CPT1 etomoxir for 1 day, both agents reduced palmitate-induced amplification by about 20–30% for all three hormones (Fig. 3G–I). The combined treatment with both etomoxir and ANT825 further lowered glucagon, insulin and somatostatin accumulation indicating distinct roles for receptor signaling and fuel metabolism in the effects of palmitate on basal glucagon and insulin secretion.

### Palmitate-induced rise in islet respiration via FFAR1-dependent and FFAR1-independent mechanisms

**Figure 4:**
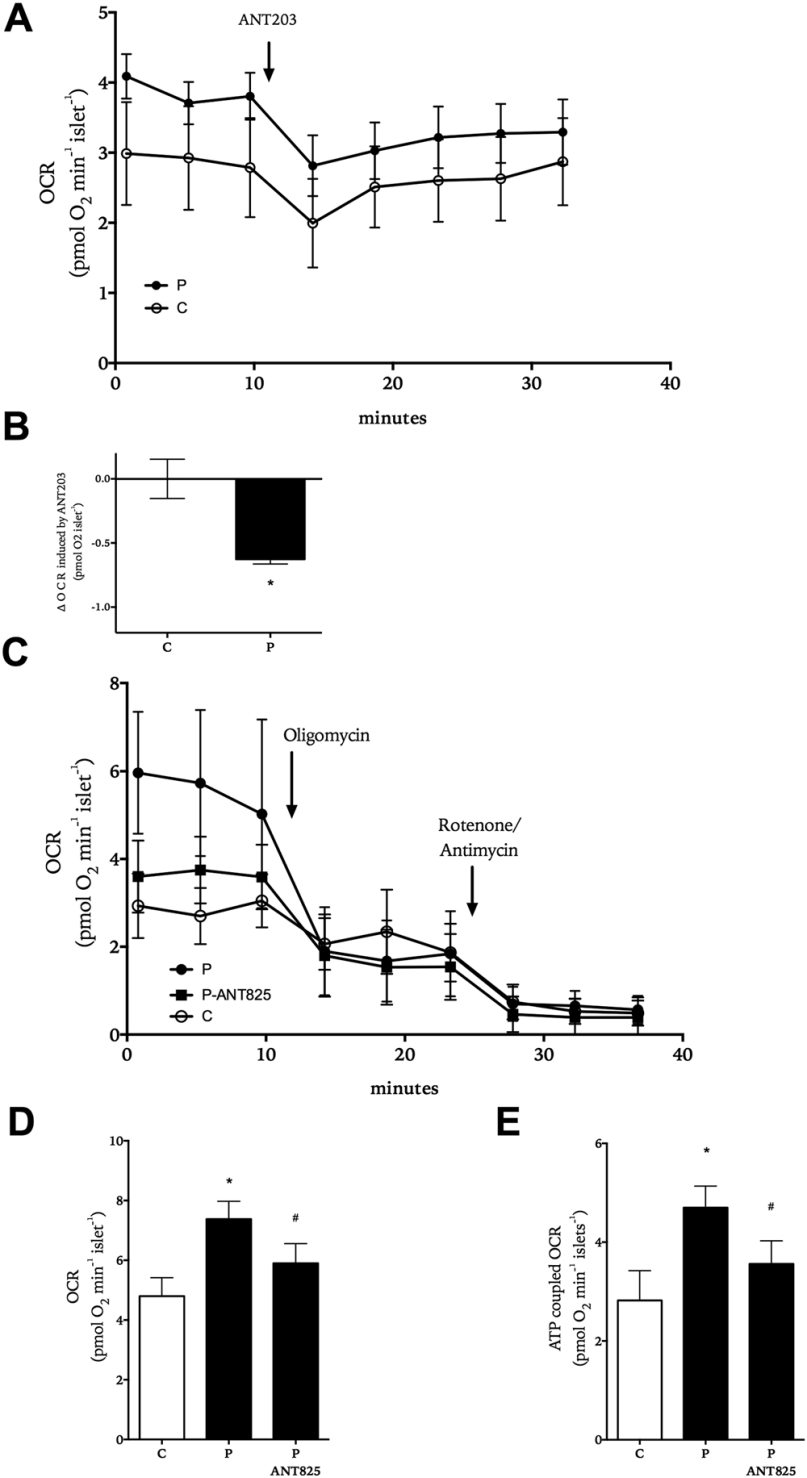
Role of FFAR1 in the effect of palmitate on human islet respiration. OCR recordings from human islets exposed to 5.6 mM glucose (white symbols) and 0.5 mM palmitate (black symbols) and sequential addition of ANT203 (panel A). Difference in OCR 20 minutes prior and post addition of ANT203 (panel B). OCR recordings from human islets exposed for 2 hours to 5.6 mM glucose (white symbols), 0.5 mM palmitate (black circles, black bars) or palmitate with ANT825 (black triangles, black bars) and the sequential addition of oligomycin and rotenone/antimycin (panel C). Mitochondrial respiration (panel D) and ATP-coupled respiration (panel E) are shown. Results show mean ± SD in (panels A−C) (N = 10) and mean ± SEM in (panels D and E) (n = 5–7 donor experiments). *P < 0.05 vs. control and ^#^p < 0.05 vs. palmitate.

Palmitate exposure at high glucose concentrations affects mitochondrial respiration and fuel metabolism of beta-cells via FFAR1-dependent and FFAR1-independent pathways^16, 26^. When our results of the present study demonstrated that palmitate affected islet hormone secretion via FFAR1 at basal glucose concentrations, we hypothesized that the fatty acid could also influence respiration at fasting glucose concentrations. Indeed, mitochondrial respiration was lowered by either acutely adding ANT203 to islets exposed to 5.6 mM glucose and palmitate (Fig. 4A,B) or including the structurally dissimilar antagonist ANT825 during culture of the islets in the presence of 5.6 mM glucose and palmitate (Fig. 4C,D). The reduction in mitochondrial respiration by ANT825 in the presence of palmitate was largely accounted for by decrease in ATP-coupled respiration (Fig. 4E).

### EndoC-βH1 human beta-cells have enhanced basal respiration and insulin secretion during palmitate exposure

**Figure 5:**
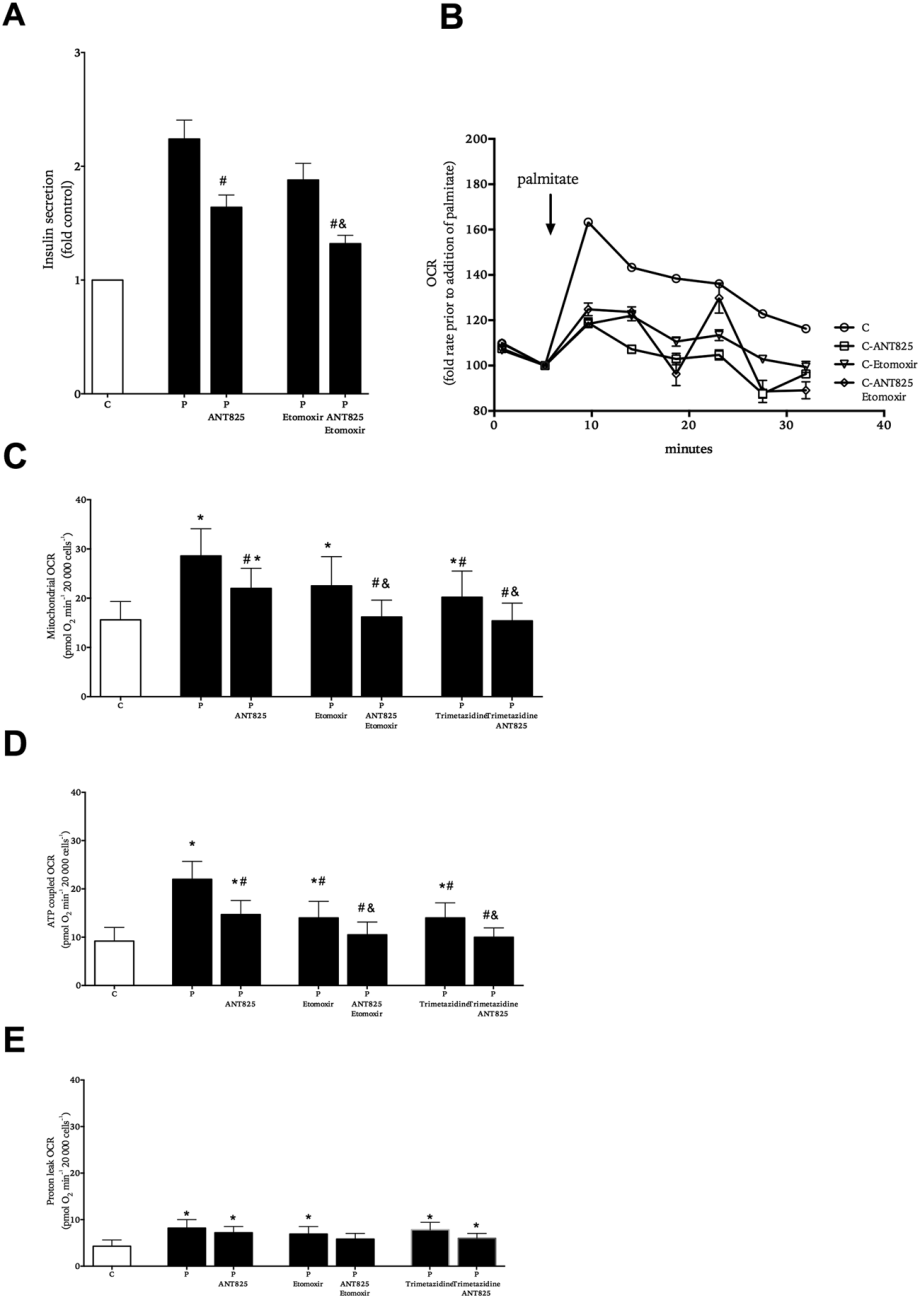
Role of FFAR1 and fatty acid β-oxidation in the effect of palmitate on EndoC-βH1 insulin secretion and respiration. Cells were exposed to 5.6 mM glucose in the absence (white symbols) or presence (black symbols) of palmitate with and without ANT825 and/or etomoxir, trimetazidine. Insulin secretion (panel A). Representative kinetic OCR recordings from cells acutely exposed to palmitate with and without ANT825 and/or etomoxir (panel B). Mitochondrial OCR (panel C), ATP-coupled OCR (panel D) and proton leak OCR (panel E). Results show mean ± SEM (panels A,C-E,) (n = 5), and mean ± SD (panel B) (N = 6). *P < 0.05 vs. control, ^#^P < 0.05 vs. palmitate alone and ^&^P < 0.05 vs. corresponding P-etomoxir/trimetazidine.

Islets contain a mixture of endocrine and non-endocrine cells. To evaluate our findings regarding islet respiration (Fig. 4) with regard to the beta-cell we used the human beta-cell line EndoC-βH1^27^. When the cells, cultured in the presence of 5.6 mM glucose, were exposed to palmitate, insulin secretion (Fig. 5A) and OCR (Fig. 5B,C) were doubled in the presence of palmitate, which was similar to what was observed in islets (Figs 1, 2 and 3). The rise in OCR was attenuated by FFAR1-antagonist ANT825 and inhibitors of fatty acid β-oxidation etomoxir and trimetazidine (Fig. 5B,C). The combination of ANT825 and the inhibitors of fatty acid β-oxidation further decreased respiration compared to either agent alone (Fig. 5C). Enhanced respiration by palmitate was mostly due to an increase in ATP-coupled respiration and antagonism of FFAR1 was mainly accounted for by reduction of ATP-coupled respiration (Fig. 5D) and had little effect on the non-ATP coupled mitochondrial respiration or proton leak, which was enhanced in cells exposed to palmitate (Fig. 5E).

## Discussion

Individuals with obesity have elevated fasting levels of both glucagon and insulin, which have been linked to the development of T2DM^1, 2^. In the present study we supply evidence that such dual islet hormonal amplification could be the result of elevated concentrations of circulating FFAs, which is characteristic of this patient group^25^. Thus, high fasting glucagon and insulin levels may be due to a combination of activation of FFAR1 and islet-cell metabolism, rather than solely compensation for insulin resistance. Understanding the mechanisms for how FFAs cause accentuated basal secretion of the islet hormones is critical to halt the aforementioned vicious circle.

We find that culture of human islets in the presence of chronically elevated palmitate concentrations simultaneously stimulates both glucagon and insulin secretion in a manner that is largely FFAR1-dependent in the early time points of culture. FFAR1 is an αq_/11_ G-protein coupled receptor that signals via phospholipase C (PLC) pathway with subsequent diacylglycerol (DAG) and inositoltrisphosphate (IP_3_) formation and protein kinase C (PKC) activity with accompanied mobilization and increase in Ca^2+^ and protein kinase D1 (PKD1) activity^15, 28, 29^. Receptor activation can be influenced by multiple allosteric binding sites and can also signal via β-arrestin recruitment to different extent^30, 31^. Stimulatory effects of fatty acids mediated by FFAR1 have been reported both for insulin^32, 33^ and glucagon secretion^17, 18, 34^. The general notion is that FFAR1 mediates its effects at elevated glucose concentrations only^32, 35^. Indeed, in a previous study we demonstrated that FFAs enhanced FFAR1 signaling, mitochondrial flux and increased glucose utilization at high glucose levels^16^. We now find that FFAR1 effects are present at 5.6 mM glucose in human islets as islets with diminished FFAR1 levels after shRNA treatment did not respond to palmitate in the same extent as islets treated with mock shRNA. Although FFAR1 mRNA and protein was around 50% less in shRNA transfected islets treated with palmitate, the decrease in secretion was on average more pronounced. This could be explained by the static incubations, where the globular shape of the islets results in the outer cells contributing more to secretion relatively and at the same time likely more affected by transfection. The effect of synthetic (non-fuel substrate) agonists was moderate in our setting, which previously studies have interpreted as FFAR1 requires elevated glucose concentrations to be activated^36^. However it can be that FFAs have the ability to both activate metabolism and FFAR1 with downstream signaling mediated by Gαq_/11_. This dual action was evident when fatty acid β-oxidation was inhibited and both respiration and hormone secretion were reduced. Indeed, when FFAR1 was competitively blocked in cells that already had less β-oxidation of palmitate, the lowering of secretion was more pronounced. In contrast, when FFAs are elevated in the presence of high glucose levels, glucose seems to be the preferential energy substrate^16^. Another possible explanation for why FFAs are more potent at stimulating secretion of glucagon and insulin at lower glucose levels compared to the synthetic agonists could be due to biased agonism at FFAR1, where a small molecule agonist was found more potent in activating the β-arrestin dependent signaling arm than the PLC-PKC-DAG signaling^30^. It is of interest and supporting of our findings that the M3 muscarinic acetylcholine receptor, which like FFAR1 also signals via the Gαq_/11_ pathway, also simultaneously stimulates both glucagon and insulin secretion^37, 38^. Also, lack of FFAR1-mediated effect at low glucose has mainly relied on studies on rodent cells, which differ from human islet cells in important aspects such as glucose transporter expression and cell-to-cell architecture, which could potentially explain why human islets might be more sensitive for FFAR1 activation at lower glucose levels^39–41^.

At later time points there was substantially higher hormone content in islets where FFAR1 had been antagonized during palmitate culture. This most likely influenced the ability of the FFAR1 antagonist to lower hormone secretion in comparison to palmitate treated islet that had much less hormone content. In addition, we have previously published that apoptosis is prevented in beta-cells cultured with palmitate when FFAR1 is antagonized, which can also impact secretion^25, 33^. Hormone release and insulin content from FFAR1 antagonized islets was still higher when exposed to palmitate compared to control conditions at the later time points, demonstrating that although FFAR1 plays an important part in elevating basal secretion, palmitate will also stimulate secretion by other means. The effect of palmitate on FFAR1 protein showed an initial rise followed by a relative decline. The increase in FFAR1 protein was minimized by co-culture with FFAR1 antagonism. The relative decline could be the result of chronic basal stimulation of the receptor and negative feedback. Other studies have demonstrated that FFAR1 protein levels can be affected by FFA culture conditions and that they are important with regards to insulin and glucagon secretion^42, 43^. Interestingly in the latter study by Del Guerra *et al*. FFAR1 protein was lower in islets from patients with T2DM.

The present study does not address if enhanced glucagon secretion precedes enhanced insulin secretion or vice versa i.e. if there is a primary hormonal derangement leading to the other. The findings place elevated FFAs as a potential primary cause for the enhanced basal hormonal secretion of the two hormones, however. Since somatostatin inhibits both insulin and glucagon secretion^44, 45^, we hypothesized that the enhanced insulin and glucagon secretion was a result of decreased somatostatin secretion. We observed that palmitate acutely lowered somatostatin secretion from human islets by about 40% during the first 30 minutes of exposure (results not shown), similar to a previous study^46^. During extended palmitate exposure elevated somatostatin secretion was observed, however. FFAR1 expression has, in addition to beta- and alpha-cells, also been identified on delta-cells^19, 34^. Our studies were on intact islets and it is therefore difficult to interpret these findings with regards to mechanism of action on the separate islet cell types. When we studied the beta-cell alone in the form of the human EndoC-βH1 cell line at 5.6 mM glucose, palmitate increased insulin secretion via FFAR1 implying that the effect of palmitate on insulin secretion is direct rather than the result of paracrine effects. We cannot rule out a paracrine effect for the palmitate-induced increase in somatostatin secretion by paracrine effects as an example insulin has been reported to stimulate somatostatin secretion from perifused chicken pancreas^45^.

As mentioned antagonizing FFAR1 in human islets could not normalize the secretion at later time points of culture, indicating an additional metabolic effect of the fatty acid. In line with this a reduction in respiration rate by etomoxir in both islets and EndoC-βH1 cells demonstrates that a large part of the increase in islet hormone release and oxygen consumption is due to utilizing the fatty acid as a substrate for ATP-production or related metabolism. Still antagonizing the receptor in the presence of palmitate had similar effects on respiration and hormone accumulation implying that the FFAR1-mediated enhancement in secretion is tightly coupled to a stimulation in mitochondrial respiration, also at fasting glucose levels. The potential mechanism for this complementary effect is that FFAR1 signaling via PLC, DAG and PKC/PKD1 leads to enhanced Ca^2+^ mobilization from intracellular Ca^2+^-stores, activation of L-type calcium-channels and enhanced islet respiration and ATP-production, which can facilitate higher secretion of both insulin and glucagon^28, 39, 46–49^. In favor of FFAR1 signaling and our previous findings with regards to the role of PKC on respiration in beta-cells^16^, these coordinated cellular events were verified concerning their effect on insulin release and mitochondrial respiration^50^. A likely scenario is that activation of FFAR1 alone at low or fasting glucose concentrations is not sufficient for maintaining increased secretion or respiration without additional substrates for the enhanced mitochondrial activity. Palmitate or long-chain fatty acids therefore act both as receptor activators and substrates for ATP-production as well as an addition to the substrate lipid pools for DAG formation.

The findings of this study come from *in vitro* experimentation and cannot directly be translated to the *in vivo* setting with its complexity involving e.g. incretins, neuronal activity and different FFAs modulating insulin and glucagon secretion. Nevertheless, the findings demonstrate a mechanism for how elevated circulating FFAs and triglycerides may lead to a concomitant increase in the basal secretion tone of glucagon and insulin secretion at fasting, which can in a chronic situation be detrimental for glucose control.

To summarize, we conclude that FFAs are regulators of not only insulin but also glucagon and somatostatin secretion at fasting glucose concentrations from human islets and that FFAR1 is crucial in this effect. This implies that the effect of FFAR1 on hormone secretion is not restricted to amplification at elevated glucose concentrations, as previously reported, but rather fuel substrate dependent. We therefore propose that the endogenous ligands of FFAR1 can, via their dual role as receptor activators and fuel metabolites, amplify release of insulin and glucagon at fasting glucose concentrations.

## Materials and Methods

### Human pancreatic islets

Human islets were obtained from brain-dead otherwise healthy individuals with no known metabolic diseases from the Uppsala University Islet Transplantation Unit and from Prodo Lab Inc. (Irvine, CA, USA). Ethical papproval for the use and for the procedures and protocols involved in handling of human islets isolated from individuals was obtained from the Regional Ethical Review Board in Uppsala, Sweden (EPN number 2010/006; 2010-02-10). All procedures involving islets were carried out in accordance with the aforementioned approval as well as in compliance with respective local guidelines and regulations. Written informed consent of all donors was given prior by individual donors or their family members and documented. The obtaining and documenting of written consent for islet donation for research was done in line with the aforementioned approval by the Regional Ethical Review Board in Uppsala, Sweden as well as according to respective local regulations and guidelines. In total, islets from 19 non-diabetic donors were used. Islets were cultured in CMRL 1066 medium containing 5.6 mM glucose and supplemented with 10% FBS (Invitrogen, Paisley, UK), hereafter referred to as control conditions. Experiments were started 3–7 days after isolation.

### EndoC-βH1 cells

EndoC-βH1 cells^27^, were grown on 1% extracellular matrix gel and 2 μg/mL fibronectin coated culture vessels in DMEM containing 5.6 mM glucose, 2% fatty acid free bovine serum albumin (BSA) fraction V (Roche Diagnostics, Mannheim, Germany), 10 mM nicotinamide, 50 μM 2-mercaptoethanol, 5.5 μg/mL transferrin, 6.7 ng/mL sodium selenite, 100 U/mL penicillin, and 100 μg/mL streptomycin. All chemicals were obtained from Sigma Aldrich if not otherwise indicated.

### Treatment of islets and EndoC-βH1 cells with long-chain fatty acid palmitate

Palmitate (sodium salt) was dissolved in 50% ethanol to a concentration of 100 mM. This stock solution was diluted in culture medium containing 0.5% BSA to 0.5 mM concentration and a molar ratio of circa 1:6 as previously described^51^. Human islets were exposed to palmitate for 6 hours as well as 1, 2, 4 or 7 days. Culture with agonists only was performed in media without FBS and at 0.1% BSA. FFAR1 antagonist ANT203 (2 μM)^33^ or ANT825 (2 μM)^16, 52, 53^ (both from AstraZeneca, Macclesfield, UK) or agonists TAK-875 (5 μM), GW9508 (5 μM) (both from Selleckchem, Munich, Germany) or TUG-499 (5 μM) (Merck KGaA, Darmstadt, Germany), or inhibitor of fatty acid β-oxidation inhibitor of carnitine-palmitoyl transferase 1 inhibitor (CPT1) etomoxir (25 μM) or 3-keto-acyl thiolase inhibitor trimetazidine (10 μM) were included or not during culture. Medium was collected and replaced at the beginning and at the end of each period and compounds such as antagonists were added to the medium freshly 30 minutes prior to addition. Oxygen consumption rate (OCR) was assessed both in islets and EndoC-βH1 cells after culture for 2 hours in the presence or absence of palmitate. Palmitate stock solution was added to minimal XF assay medium (Agilent Technologies, Santa Clara, CA, USA) containing 0.5% BSA with the final concentration of 0.5 mM. For acute addition of palmitate during OCR measurements 1 mM palmitate and 1% BSA minimal XF assay media was injected to assay media with the final concentration of 0.1 mM palmitate and 0.1% BSA. FFAR1 antagonist ANT203 (2 μM)^33^ or ANT825 (2 μM)^16, 52, 53^ inhibitor of fatty acid β-oxidation inhibitor of carnitine-palmitoyl transferase 1 inhibitor (CPT1) etomoxir (25 μM) or 3-keto-acyl thiolase inhibitor trimetazidine (10 μM) were included or not during culture. All chemicals were obtained from Sigma Aldrich if not otherwise indicated.

### Hormone secretion and content measurements

Glucagon, insulin (Mercodia AB, Uppsala Sweden) and somatostatin (Peninsula labs, San Carlos, CA, USA) concentrations in culture media and cell lysate samples were measured by ELISA. Hormone content was normalized to total protein content as measured by the DC protein assay (Bio-Rad Laboratories, Hercules, CA, USA) and then presented as fold of control treatment.

### FFAR1 shRNA down-regulation

The short hairpin RNA (shRNA) of FFAR1 was used to inhibit the expression of FFAR1 and was administered with lenti-viral transduction particles SHCLNV VSV-G (Mission transduction particles, Sigma Aldrich) containing shRNA targeting Ffar1/Gpr40 with the sequence CCGGCGCCTCCAACGTGGCCAGCTTCTCGAGAAGCTGGCCACGTTGGAGGCGTTTTT and non-mammalian control for comparison. About 50 islets were transduced in each well (non-tissue culture 96-well plate) with 2–5 × 10^6^ TU of transduction particles in 50 μL of 1:1 mixture of DMEM mixed with CMRL culture medium (no FBS no BSA) for 2 hours as previously described^54^. Additional 150 μL culture medium was then added and allowed to incubate for 22 hours. Subsequently medium was replaced with 200 μL CMRL medium. Based on estimated receptor turnover treatment was started 3 days after initiation of transfection and samples collected 2 days after start of palmitate treatment.

### Measurement of FFAR1 mRNA level by real time PCR

Total mRNA was isolated from islet cells using NucleoSpin® RNA (Macherey-Nagel, Duren, Germany) and reversely transcribed into cDNA with SuperScript™ III First-Strand Synthesis System for RT-qPCR (Invitrogen, Carlsbad, CA, USA). The real-time PCR was performed in 10 μL volume using Dynamo Capillary SYBR Green qPCR kit (Thermo Scientific, Waltham, MA, USA). The following primers were used for amplification: FFAR1 (forward primer, 5′-ATCACAGCCTTCTGCTAC and reverse primer, 5′-CCTAGATTGGGGTACAGG), β-actin (forward primer, 5′-ACGTGGACATCCGCAAAGAC) and (reverse primer, 5′-CAGGGCAGTGATCTCCTTCT). FFAR1 mRNA level was normalized to the reference gene β-actin using the following formula: target amount = 2−ΔΔCt, where ΔΔCt = [Ct (GPR40 KO) − Ct (β-actin KO)] − [Ct (GPR40 control) − Ct (β-actin control)]^55^.

### Measurement of FFAR1 protein level by ELISA

Around 100–500 isolated human islets were collected after culture and washed three times with ice cold PBS. Cells were lysed in 100 μl of 1% TritonX-100 (Sigma-Aldrich) in PBS. Lysates underwent five quick freeze and thaw cycles to enhance amount of membrane bound proteins. Total FFAR1 amount in lysate samples was measured by a commercial human FFAR1/GPR40 Sandwich ELISA (LS-F8454, LifeSpan BioSciences, WA, USA) according to the manufacturer’s instructions. HEK293 cells were used as a negative control and showed sparse reactivity in the assay in comparison to EndoC-βH1 cells (see Supplementary Fig. S1).

### Cellular respiration

Mitochondrial respiration of isolated human islets and EndoC-βH1 cells was determined by measuring OCR in the Extracellular Flux Analyzer XF96e (Agilent Technologies). Assays were performed in minimal XF assay medium (Agilent Technologies) set to pH 7.4 and supplemented with 5.6 mM glucose. Both islets (10–30 per well, and 10 wells for each condition (N = 10)) and EndoC-βH1 cells were placed in 96-well (60000 cells per well, and at least 6 wells for each condition (N = 6)) precoated (see above) culture plates. Mitochondrial function was determined by measuring basal respiration, ATP-coupled respiration, proton leak, maximal respiratory capacity by adding inhibitors of the electron transport chain oligomycin (2 μM), rotenone (5 μM) and antimycin (5 μM) as well as the ionophore FCCP (2 μM), as previously described^16^. Basal OCR was measured following a 2-hour culture. All OCR measurements were corrected for non-mitochondrial OCR. All chemicals were obtained from Sigma Aldrich if not otherwise indicated.

### Statistical analysis

Analysis was done with Graph Pad Prism software, version 6 (San Diego, CA, USA). Paired t-test and repeated measures one-way ANOVA (with Fishers LSD post-hoc test) were used for statistical analysis. P < 0.05 was considered statistically significant.

### Data availability

The datasets generated during and/or analyzed during the current study are available from the corresponding author on reasonable request.

## Electronic supplementary material

Supplementary Figure S1
