# Supplementary material for: Basal hypersecretion of glucagon and insulin from palmitate-exposed human islets depends on FFAR1 but not decreased somatostatin secretion
Source: Sci Rep. 2017 Jul 5;7:4657. doi: 10.1038/s41598-017-04730-5 (PMC5498543; doi:10.1038/s41598-017-04730-5)
Supplement: Supplementary file 1 — Supplementary Figure S1 [file 41598_2017_4730_MOESM1_ESM.pdf]

Basal hypersecretion of glucagon and insulin from palmitate-exposed human islets depends on FFAR1 but not decreased somatostatin secretion

[1]Kristinsson H[*], [1]Sargsyan E, [1]Manell H, [2]Smith DM, [3]Göpel SO and [1]Bergsten P

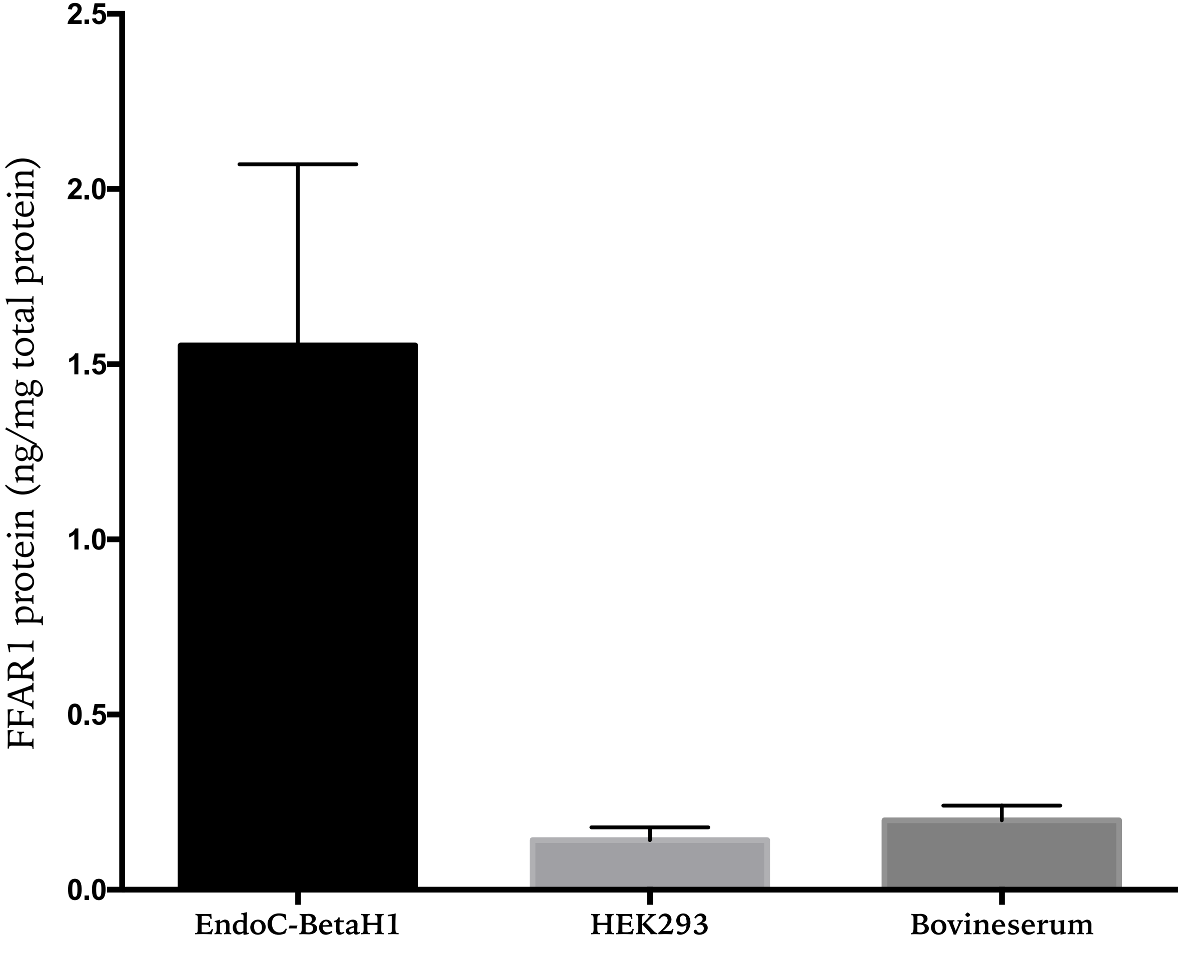

Supplementary Figure S1. Negative control for FFAR1 protein assay. FFAR1 protein reactivity from lysates of EndoC-betaH1 beta cells, HEK293 cells and from concentrated bovine serum.
